# Analyzing willingness for extracorporeal cardiopulmonary resuscitation in refractory ventricular fibrillation

**DOI:** 10.1371/journal.pone.0281092

**Published:** 2023-01-26

**Authors:** Seon Koo Kim, Ju Ok Park, Hang A. Park, Choung Ah Lee, Sola Kim, Soon-Joo Wang, Hye Ji Park, Hye Ah Lee

**Affiliations:** 1 Department of Emergency Medicine, Hallym University Dongtan Sacred Heart Hospital, Gyeonggi-do, Republic of Korea; 2 Clinical Trial Center, Ewha Womans University Mokdong Hospital, Seoul, Republic of Korea; Fondazione IRCCS Policlinico San Matteo, ITALY

## Abstract

Extracorporeal cardiopulmonary resuscitation (ECPR) for refractory ventricular fibrillation/ventricular tachycardia in out-of-hospital cardiac arrest has recently been recommended for selected patients with favorable prognostic features. We aimed to identify factors affecting the willingness of emergency physicians to implement extracorporeal cardiopulmonary resuscitation (ECPR). We conducted a factorial survey with nine experimental vignettes by combining three different scene time intervals and transportation time intervals. Emergency physicians reported willingness to implement ECPR (1–100 points). Respondent characteristics that could affect the willingness were studied. Multilevel analysis of vignettes and respondent factors was conducted using a mixed-effects regression model. We obtained 486 vignette responses from 54 emergency physicians. In the case of longer scene time intervals, there was a significant difference in the willingness scores at 9 and 12 min transportation time intervals. When the pre-hospital time interval was > 40 min, emergency physicians demonstrated lower willingness to implement ECPR. Clinical experience of 15–19 years showed a significant favorable effect on willingness to implement extracorporeal membrane oxygenation (ECMO). However, the mean willingness scores of EPs for ECMO implementation were more than 75 across all vignettes. In ECPR, the prehospital time interval is an important factor, and the willingness of emergency physicians to implement ECMO could be mutually affected by scene time intervals, transportation time intervals, and total prehospital time.

## Introduction

The annual global incidence of out-of-hospital cardiac arrest (OHCA) in adults is 62/100 000, of which 75–85% are of cardiac origin [1]. Although ventricular fibrillation/ventricular tachycardia (VF/VT) is a favorable prognostic marker for OHCA, half of the patients with VF/VT OHCA are refractory to treatment and fail to achieve a sustained return of spontaneous circulation after the recommended management [2]. In particular, refractory VF/VT is defined as resistance to at least three defibrillations, 3 mg of epinephrine, and 300 mg of amiodarone, and failure to achieve a return of spontaneous circulation after cardiopulmonary resuscitation (CPR) for more than 10 min [3]. Multiple case series have demonstrated the potential benefits of mechanical circulatory support, including extracorporeal cardiopulmonary resuscitation (ECPR) in some selected cases of OHCA [4,5]. The use of ECPR is described in numerous facilities with impressive results in appropriately selected patients, such as a neurologically intact survival of over 30% in patients with refractory OHCA [6–8]. In 2015, the Minnesota Resuscitation Consortium implemented an advanced ECPR protocol for refractory VF/VT, wherein 50% of patients were discharged from the hospital with good neurological function [7].

However, in the 2020 update of American Heart Association Guidelines on adult resuscitation, it was stated that further research is needed to define patients who would benefit most from the intervention [4]. Moreover, there is still insufficient evidence on whether emergency medical technicians should provide multiple defibrillation attempts in refractory VF/VT, or other alternative treatments should be attempted by early transfer to the hospital [9]. Many countries and regions may not have local emergency medical services (EMS) guidelines for ECPR activation. Despite the recommendation of providing on-site active CPR for all patients undergoing cardiac arrest [10], prehospital healthcare providers struggle to balance active on-site treatment and hospital transfer for patients with refractory VF/VT. Few studies have investigated the optimal time to consider alternative resuscitation methods in patients with OHCA who received prolonged CPR [11–13].The results for the optimal time interval from collapse to ECPR initiation differed among studies, although survivors usually had a significantly short time interval. The duration of ischemia (collapse to ECPR) was vital for survival according to previous studies [14].

Further, this lack or variability of evidence can affect the decision of the doctor to implement ECPR for patients with refractory VF/VT. Well-designed prospective studies evaluating the effectiveness of ECPR are challenging in practice. Controlling various EMS variables by region and country is necessary for conducting randomized controlled trials; moreover, managing EMS conditions in the real world may result in several problems, including ethical issues. There has been one small randomized controlled trial on the use of ECPR for OHCA [15], and several available studies are observational [4]. A recent scoping review regarding the effects of ECPR in patients with refractory cardiac arrest showed that only six studies compared ECPR and traditional CPR for OHCA and that current knowledge is mainly drawn from single-center observational studies, with no randomized controlled trials included [16]. Moreover, ECPR may not be easily attempted by inexperienced physicians as it requires many resources such as trained personnel and critical care facilities for implementation and maintenance.

A vignette survey could be used for investigating respondent beliefs, attitudes, or judgments [17]. This method uses true-to-life vignettes presented to a decision-maker who is asked to judge a familiar situation [18]. The vignette survey can allow simultaneous presentation of several explanatory and contextual factors and the estimation of unconfounded and context-dependent effects of vignette factors. Depending on the research question, vignettes can be presented to respondents in different forms, such as text vignettes, cartoons, pictures, audio, or video vignettes [17].

Thus, using simulated vignettes, we attempted to analyze prehospital time factors that emergency physicians (EPs) consider before implementing ECPR in patients with refractory VF/VT when confounders were controlled.

## Materials and methods

### Study design

We used a factorial analysis approach with an experimental vignette design to explore specific attitudes bounded by clinical decision-making [17,19–21].

### Ethical statement

The study was approved by the Research Ethics Committee of Hallym University Dongtan Sacred Heart Hospital (HDT 2021-01-013-001). Electronic written informed consent was provided as a part of the online survey via email.

### Experimental vignettes

The experimental vignettes were outlined during the brainstorming session with clinical experiences of the authors and were developed based on an extensive background literature review of ECPR. In particular, the prehospital time in the vignettes was based on previous observational studies in Korea [11,22–24]. After brainstorming, vignettes drafted by one author (SK), who was a five-year experienced EP, were preliminarily reviewed by the co-authors (JP, HP, CL, SK) and two other experienced EPs (more than five years of clinical experience) who were not involved in the study. For each vignette, they reviewed the patient’s story for realism, confounders not worthy of the study objectives, and typos or inscriptions. In the preliminary review, differences in willingness for each vignette were not compared. After preliminary review, vignettes were then polished for relevance and clarity. Therefore, experienced EPs were engaged throughout the development processes of the vignettes.

In all the vignettes, it was assumed that the doctor was working in the emergency department where the ECPR team was always available, and the doctor received a call from the paramedics who had just left the scene. The paramedics summarized the patient characteristics and prehospital care and notified that they would arrive after a specific transportation time interval (TTI). We used an experimental design, with the factors of three different scene time intervals (STIs) (11, 19, and 23 min) and three different TTIs (9, 12, and 16 min) to determine prehospital time intervals (PTIs). The full factorial vignettes of two factors with three levels each resulted in 3×3 = 9 different vignettes. The other factors were controlled within the vignettes in terms of activation time interval (ATI), response time interval (RTI), the level of prehospital care, and patient characteristics as long as the vignettes resembled actual situations and felt like different situations each. All vignettes of OHCA were witnessed, received bystander CPR, and were defibrillated by witnesses or paramedics. The ATI and RTI were the same, and paramedics only performed basic life support (Table 1).

**Table 1:** Factors and variables as described in the vignettes.

Factors	Variables	Variable levels
Patient	Age (years)	55±3
	Sex	Male
	Place of cardiac arrest	Home or public places
Prehospital care	Witness	Yes
	Bystander CPR	Yes
	Defibrillation in prehospital care	3–4 times
	Level of care	Basic life support only
Prehospital time	Activation time interval	Immediate
	Response time interval	6 min
	Scene time interval	1) 11 min in 3 vignettes
		2) 19 min in 3 vignettes
		3) 23 min in 3 vignettes
	Transportation time interval	1) 9 min in 3 vignettes
		2) 12 min in 3 vignettes
		3) 16 min in 3 vignettes

CPR, Cardiopulmonary resuscitation.

Participants could convey their willingness to activate an ECPR team to apply ECMO using a scale of 0 to 100, with 0 indicating no willingness and 100 indicating complete willingness after reading each vignette. Further, we collected characteristics of participants such as age, sex, years of clinically active career, and ECPR experiences (S1 File).

### Participants

Convenient sampling was conducted for EPs with more than 5 years of clinical experience in the emergency department. We sent research explanations and consent forms to EPs whose e-mail addresses were disclosed among the EPs participating in OHCA-related research consortiums or those working as EMS directors; EPs who agreed to participate were included. The target sample size for repeated-measures analysis of variance for nine blocks was 22 (power 0·95, α-error 0·05); however, the survey was delivered to 312 EPs.

### Procedure

The data collection was conducted in March 2021 using the electronic survey form. Two reminders were sent to the participants for 3 weeks until the target sample size was reached. All participants responded to each of the nine vignettes independently using the online link of the survey submission form sent to individual e-mail. They couldn’t know each other’s answers. The order of the vignettes was randomized to prevent the effects of autocorrelation. They were restricted from returning to previous vignettes or skipping without answering the current one during the process.

### Variables and statistical analysis

The primary unit of analysis was a vignette. The dependent variable was the willingness to implement extracorporeal membrane oxygenation (ECMO) for each vignette patient. The independent variables were patients’ characteristics described in each vignette and participants’ characteristics. Descriptive statistics were used to summarize the participants’ characteristics. The dependent variable was squared to ensure that the normal distribution resolved the left skewness. The t-test and one-way analysis of variance were used, and the statistical results showed a quadratic mean and 95% confidence interval (CI).

We obtained balanced experimental vignette data and multiple measurements clustered within the participant as each participant contributed nine vignette responses. The data had a multilevel structure of both vignette factors and participant characteristics; thus, we applied multilevel analysis using a mixed-effects regression model fitted with vignette factors as level 1 and participant factors as level 2. The balanced factorial design allowed the unconfounded estimation of all main effects [25], and we included only two factors in the vignettes, STI and TTI. We conducted an iterative model-building process with a bottom-up strategy—from an unconditional, null model and then added level 1 and 2 covariates sequentially. Owing to the interaction between STI and TTI, we could not show their effects independently. Instead of STI and TTI, we used the PTI, which is the sum of ATI, STI, and TTI, to present the effect of time factor on the willingness to implement ECMO. Likelihood ratio tests, Akaike information criterion, and Bayesian information criterion were used to measure the goodness of fit. To test the willingness score between the vignettes, a post hoc test was conducted by comparing the difference in the adjusted means using Bonferroni’s method.

## Results

We collected 486 responses from 54 EPs for nine vignettes. Table 2 shows the willingness for ECMO application according to the characteristics of the EPs. The mean willingness score to implement ECMO was 82.0 (95% CI 80.5–83.6). EPs with no experience in ECMO implementation showed a higher willingness than those who were experienced; however, the difference was not statistically significant (83.9 vs. 80.9, *p* = 0.0705). EPs with a 15–19-year-long career indicated the highest willingness for ECMO implementation (84.9, 95% CI 82.69–87.03), whereas EPs with < 10 year-long career exhibited the lowest willingness for ECMO application (69.5, 95% CI 61.94–76.26) (*p* < 0.001). The sex and age of EPs and ECMO availability in the current working hospital were not associated with the willingness to implement ECMO.

**Table 2:** The willingness scores for ECMO application according to the characteristics of the Eps.

Characteristics		Respondents		Willingness to implement ECPR		*P*
		Total = 54		(1–100)		
		N	%	Mean	95% CI	
Age (years old)	< 40	11	20.4	79.7	(75.99, 83.27)	0.2189
	40–49	35	64.8	82.2	(80.20, 84.16)	
	≥50	8	14.8	84.3	(81.23, 87.29)	
Sex	Male	38	70.4	82.1	(80.20, 83.91)	0.9137
	Female	16	29.6	81.9	(79.02, 84.66)	
Clinical career (years)	< 10	4	7.4	69.5	(61.94, 76.26)	< 0.001
	10–14	12	22.2	80.0	(76.57, 83.23)	
	15–19	23	42.6	84.9	(82.69, 87.03)	
	≥ 20	15	27.8	82.2	(79.29, 85.10)	
Experience of ECMO implementation	Yes	34	63.0	80.9	(78.89, 82.90)	0.0705
	No	20	37.0	83.9	(81.41, 86.23)	
ECMO availability in current working hospital	Yes	28	51.9	80.2	(77.91, 82.34)	0.0599
	No	26	48.2	84.0	(81.82, 86.09)	

ECMO, Extracorporeal membrane oxygenation; EMPs, Emergency medicine physicians; N, number;95% CI, 95% Confidence interval.

The willingness score for ECMO implementation decreased as TTI and PTI increased, although the difference was statistically insignificant. However, the willingness score for ECMO implementation significantly decreased as STI increased (*p* = 0.0288) (Table 3).

**Table 3:** The willingness scores for ECMO application according to the prehospital time factors of vignettes.

		Willingness to implement ECPR		*P*-value
		Mean	95% CI	
STI (min)	11	84.3	(81.80, 86.66)	0.0288
	19	82.5	(79.88, 85.07)	
	23	79.2	(76.17, 82.11)	
TTI (min)	9	83.9	(81.33, 86.39)	0.2189
	12	81.4	(78.59, 84.13)	
	16	80.7	(77.94, 83.40)	
PTI (min)	20	85.7	(81.59, 89.55)	0.1866
	23	84.7	(80.48, 88.78)	
	27	82.4	(77.69, 86.79)	
	28	83.2	(78.65, 87.49)	
	31	82.9	(78.17, 87.33)	
	32	82.8	(77.90, 87.45)	
	35	79.0	(75.30, 82.46)	
	39	78.3	(72.98, 83.21)	

ECMO, Extracorporeal membrane oxygenation; 95% CI, 95% Confidence interval; STI, Scene time interval; TTI, Transportation time interval; PTI, Prehospital time interval.

**Fig 1:**
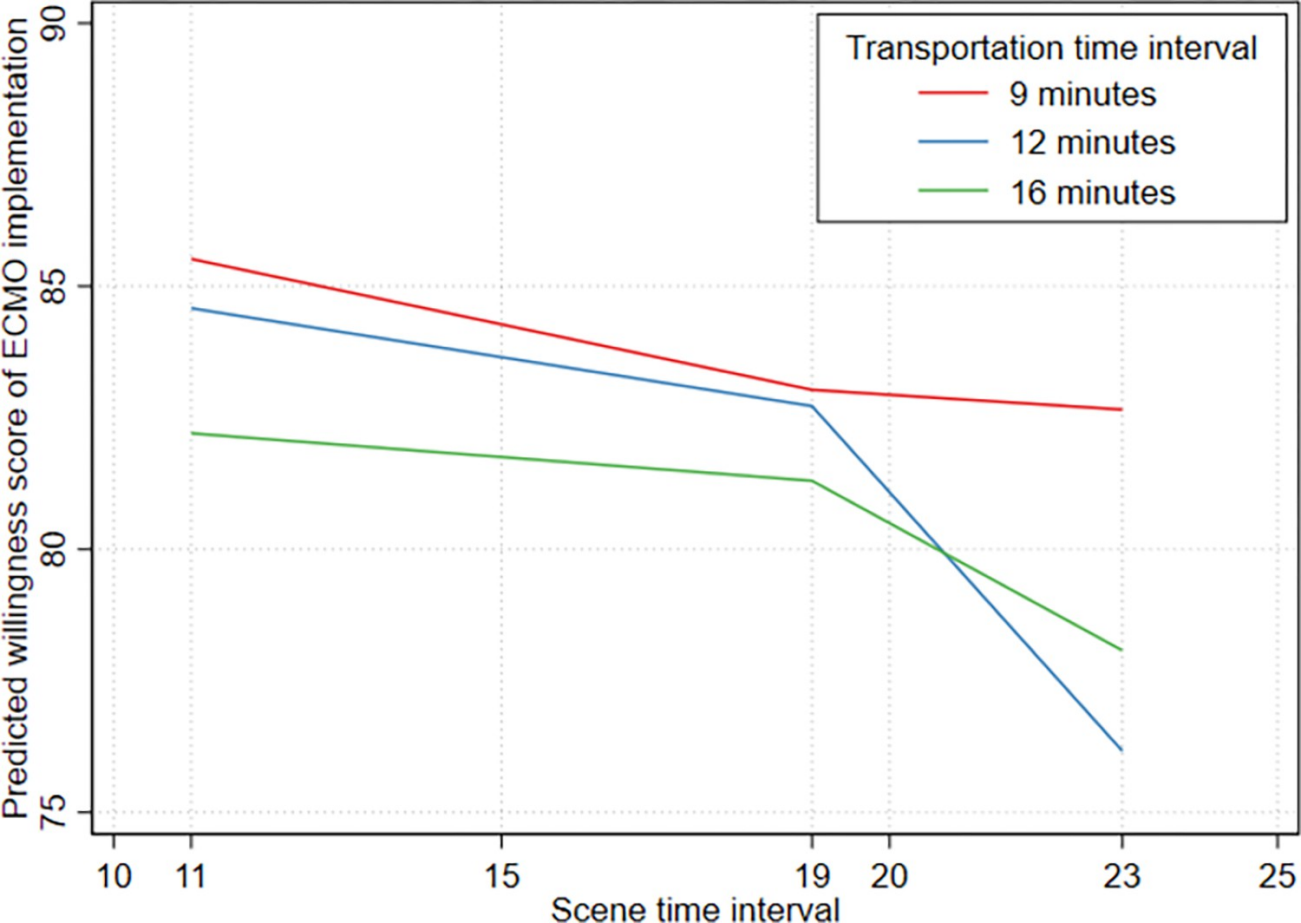
The interaction between scene time interval and transportation time interval.

A mixed-effects regression analysis was conducted for the 486 responses. The intraclass correlation of the null model (random intercept only model, null model) was 0·75 (95% CI 0.66–82.0), and the likelihood ratio test between the fixed and random intercept model was found to be significant (*Χ*^2^ = 489.35, *p* < 0.001), confirming a better fit with a mixed-effects model approach. Adding vignette factor variables (level 1), STI and TTI, to the null model (model 1) increased the intraclass correlation to 0·77 (95% CI 0.68–0.83). The likelihood ratio test indicated a significant improvement in the model fit (*Χ*^2^ = 516.29, *p* < 0.001). The Akaike information criterion and Bayesian information criterion in model 1 were 8478.35 and 8524.40, respectively. Adding the EP level variables (level 2) to model 1 (i.e., model 2), the fitness of the model was slightly disproved (*Χ*^2^ = 493.71, *p* < 0.001). The Akaike information criterion and Bayesian information criterion were 8377.14 and 8452.49, respectively. Further, significant interaction between STI and TTI was observed (*F* [8, 11] = 6.64, *p* = 0.0027) in model 1, and this interaction was persistently significant after adjustment for the EP factors (model 2) (*F* [8, 22] = 6.64, *p* = 0.0002) (Fig 1).

**Fig 2:**
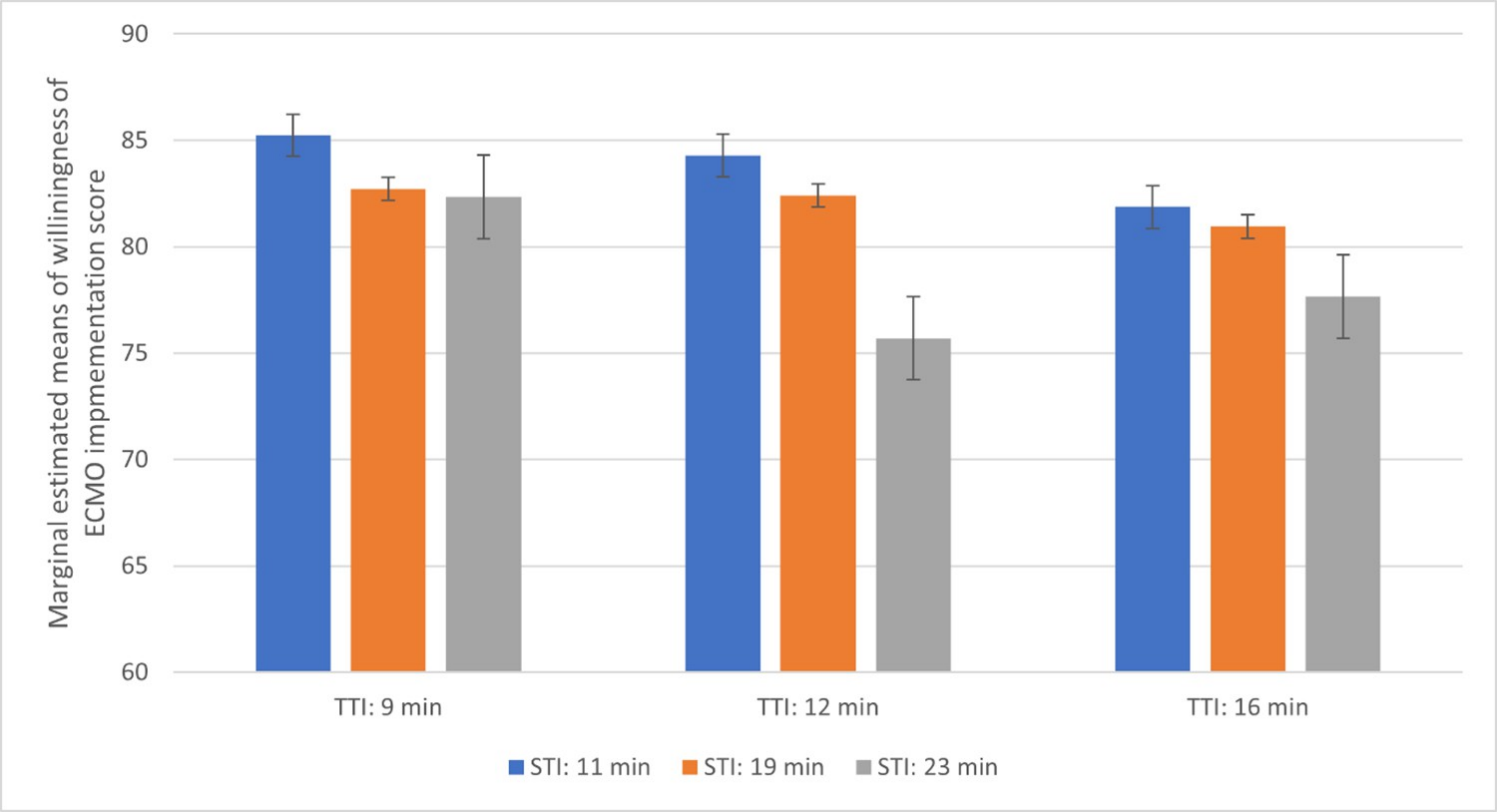
Analysis of estimated marginal means test and post hoc Bonferroni-adjusted pairwise comparison for prehospital time factors across vignettes. STI: Scene time interval; TTI: Transportation time interval; *p*-values from the post hoc Bonferroni adjusted pairwise comparison.

To reveal the difference in willingness score in the interaction between STI and TTI, the estimated marginal means were calculated and the Bonferroni-adjusted pairwise comparison for multiple comparisons was performed (Table 4 and Fig 2). The vignette with the lowest mean willingness score was vignette 6, with an STI of 23 min and a TTI of 12 min (75.7, 95% CI 70.66–80.77). Fig 2 visually presents the estimated mean willingness score for each vignette by grouping it using TTI. Overall, if vignettes had the same TTI, a longer STI implied a lower willingness score. In the post hoc test, between vignettes 3 and 6 (TTI 9 vs. 12 min at same STI 23 min), vignettes 4 and 6 (STI 11 vs. 23 min at same TTI 12 min), and vignettes 5 and 6 (STI 19 vs. 23 min at same TTI 12 min), the willingness score for ECMO implementation was significantly different.

**Table 4:** Analysis of estimated marginal means of willingness score for ECMO implementation for each vignette.

Number of vignettes	Factors of vignettes		Marginal estimated means		
	STI (min)	TTI (min)	Estimated	Standard error	95% CI
1	11	9	85.2	2.24	(80.86, 89.63)
2	19	9	82.7	2.31	(78.18, 87.26)
3	23	9	82.3	2.33	(77.78, 86.90)
4	11	12	84.3	2.27	(79.85, 88.74)
5	19	12	82.4	2.33	(77.85, 86.96)
6	23	12	75.7	2.58	(70.66, 80.77)
7	11	16	81.9	2.34	(77.29, 86.47)
8	19	16	81.0	2.37	(76.31, 85.61)
9	23	16	77.7	2.50	(72.78, 82.56)

ECMO, extracorporeal membrane oxygenation; STI, scene time interval; TTI, transportation time interval; 95% CI, 95% confidence interval.

**Fig 3:**
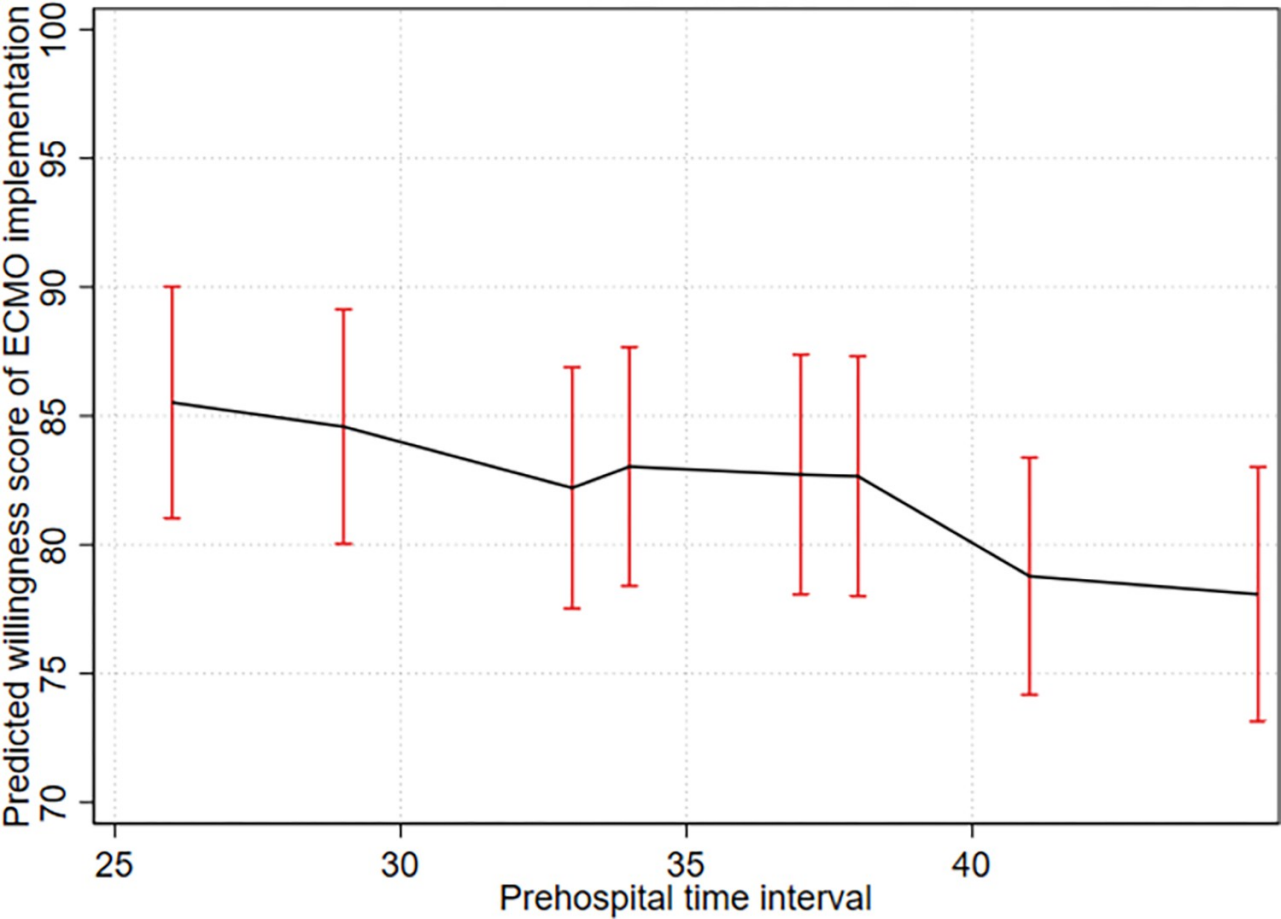
Predicted willingness score for extracorporeal membrane oxygenation (ECMO) implementation based on prehospital time interval.

Table 5 presents the results of the multilevel mixed-effects regression. For PTI, the coefficient was significant at 29, 41, and 45 min. The effect on the willingness score for ECMO implementation was significant with longer PTIs but wide 95% CIs. Among the characteristics of EPs, 15-19-year-long careers showed a significant effect on the willingness to implement ECMO compared to less than 10 year-long career. The predicted willingness score to implement ECPR is shown in Fig 3. When the PTI was 26 min, the predicted score was 85.5 (95% CI 81.02–90.02); however, at 41 min, it significantly decreased to 78.8 (95% CI 74.17–83.38) (the difference was -6.74, *p* < 0.001 after adjustment by Bonferroni method). At 45 min, it significantly decreased to 78.1 point (the difference was -7.44, *p* < 0.001 after adjustment by Bonferroni method).

**Table 5:** Effect of prehospital time and EP characteristics on the willingness score for ECMO.

					Null		Model 1		Model 2	
					Β	95% CI	β	95% CI	β	95% CI
*Fixed effect*										
Intercept		6727.2	(6054.05, 7400.39)		7338.1	(6581.21, 8094.94)		4088.9	(-189.52, 8367.42)	
Level 1: Vignettes										
Prehospital time Interval	26 min				*Reference*			*Reference*		
	29 min				-158.9	(-677.80, 360.06)		-158.9	(-677.80, 360.06)	
	33 min				-553.7	(-1072.67, -34.81)		-553.7	(-1072.67, -34.81)	
	34 min				-417.9	(-936.78, 101.08)		-417.9	(-936.78, 101.08)	
	37 min				-468.9	(-987.86, 50.00)		-468.9	(-987.86, 50.00)	
	38 min				-479.6	(-998.56, 39.30)		-479.6	(-998.56, 39.30)	
	41 min				-1103.1	(-1552.55, -653.74)		-1103.1	(-1552.55, -653.74)	
	45 min				-1212.4	(-1731.30, -693.44)		-1212.4	(-1731.30, -693.44)	
Level 2: EMP characteristics										
Age	< 40 years old							*Reference*		
	40–49 years old							-1572.4	(-4168.20, 1023.42)	
	≥50 years old							-873.6	(-4572.75, 2825.52)	
Sex	Male							*Reference*		
	female							612.7	(-1125.01, 2350.47)	
Career	< 10 years							*Reference*		
	10–14 years							2784.4	(-825.22, 6393.96)	
	15–19 years							4262.8	(232.42, 8293.17)	
	≥ 20							3554.9	(-969.73, 8079.48)	
Experience	Yes							*Reference*		
	No							490.2	(-951.75, 1932.16)	
*Random effect*		Variance	95% CI		Variance	95% CI		Variance	95% CI	
Intercept		6142312	(4138426, 9116508)		6159763	(4154847, 9132149)		6338929	(4155556, 9669470)	
*Model fit*		χ^2^	AIC	BIC	χ^2^	AIC	BIC	χ^2^	AIC	BIC
		489.35 (*p* < 0.001)	8613.73	8626.29	510.59 (*p* < 0.001)	8498.67	8540.53	488.11 (*p* < 0.001)	8397.46	8468.62

EMP, Emergency medicine physicians; ECMO, Extracorporeal membrane oxygenation; CI, Confidence interval; AIC, Akaike information criterion; BIC, Bayesian information criterion.

## Discussion

This study revealed that after adjustment for all patient characteristics, the willingness score of EPs for ECMO implementation decreased as the prehospital time increased. In particular, the willingness of EPs significantly decreased when the PTI was > 40 min. Depending on the EMS system, either the STI or TTI may be modified while determining the PTI for ECPR application. Therefore, we separately analyzed STI and TTI. In case of longer STI (23 min), the willingness scores were significantly different at TTIs of 9 and 12 min. Among the characteristics of EPs, a clinical career in the emergency department showed a significant effect. EPs with a 15–19-year-long career displayed a significantly higher willingness than those with a < 10 year-long career; in contrast, EPs with a > 19 year-long career did not exhibit such a difference. However, the mean willingness scores of EPs for ECMO implementation were more than 75 across all vignettes, meaning most EPs are confident of the outcome following ECPR.

Essentially, the criteria for prehospital ischemic time to select ECPR candidates should be objectively determined from data collected from randomized trials. In the recent single-center randomized clinical trial in Prague, the bundle of early transport, ECPR and invasive assessment in refractory OHCA did not significantly improve 180-day survival with favorable neurologic outcome compared with standard care [26]. However, this trial was terminated because of reaching a stopping rule within prespecified criteria for futility, so the trial was possibly underpowered to detect a clinically relevant difference. Similarly, many similar trials could be hard to conduct powerful RCT in the future, and we should expect the meta-analysis to combine those randomized trials.

Particularly, in Korea, where this study was conducted, the national health insurance would reimburse ECMO for reversible cardiac arrest (accidental hypothermia, drug intoxication) or patients likely to be resuscitated by performing CPR, i.e., cardiac arrests where the onset can be inferred with relative accuracy or that has been witnessed. In Korea, National Health Insurance (NHI) does not reimburse the cost of ECMO when 1) CPR is performed for more than 60 min without sufficient tissue perfusion, or 2) cardiac arrest is not witnessed, so it is not possible to confirm the time of the cardiac arrest. It is conditional reimbursement because it is hard to prove the sufficient tissue perfusion during CPR or to predict the probability of survival after resuscitation when EPs decide to implement the ECPR. Especially if the patient was not resuscitated after ECPR, it is more difficult to get the reimbursement from NHI and may have a significant economic burden on a patient’s family. EPs should explain this condition to the patient’s family and might be affected by this NHI guideline when they decide on the implementation of ECPR for patients with refractory VF/VT. In this situation, the prehospital time interval could be an important consideration.

ECPR is a complex intervention requiring a highly trained team, specialized equipment, and multidisciplinary support within the healthcare system [4]. Furthermore, ECPR initiation involves a multidisciplinary approach, and it is highly institution-dependent. The experimental vignette method can enable the estimation of unconfounded and context-dependent effects of explanatory vignette factors. [17]. Therefore, we assumed in the survey that there was no institutional limitation for all vignettes to remove the potential confounder to minimize the influences of respondent’s experience or working environment on their opinions.

The ischemic time from collapse to ECPR is a known factor related to the outcome of patient with refractory VT/VF following ECPR [14]. The inclusion criteria for ECPR in adults recommended by Extracorporeal Life Support Organization (ELSO) recently included the time from arrest to first CPR < 5 minutes and the time from arrest to ECMO < 60 minutes [27]. Several studies have reported the association of shorter prehospital time intervals with survival and good neurological outcomes after ECPR [28–35]. The difference in willingness as per prehospital time interval is not surprising because doctors decide to implement ECPR knowing its relevance. However, the prehospital time interval, which could predict a good prognosis in previous observational studies, showed inconsistent results, ranging from 15 to 40 min in different studies. Therefore, the optimal prehospital ischemic time for administering ECPR cannot be determined as an absolute value. According to Reynolds et al., the probability of survival with good neurological outcomes decreases after 16 min of CPR [36]. Kim et al. suggested that the optimal cut-off time to switch from conventional CPR to ECPR is 21 min [11]. A previous study reported that continuing CPR for more than 15 min on the scene was associated with a reduced probability of survival with good neurological outcomes in patients with refractory OHCA [37]. Additionally, survivors with refractory OHCA had a significantly shorter time interval from collapse to ECMO initiation (40 [25–51] min vs. 54 [34–74] min, *p* = 0·002) and a higher rate of intra-arrest percutaneous coronary intervention (88% vs. 70%; *p* = 0·04) than non-survivors [14]. Consistent with these findings, our study showed that longer prehospital time duration was associated with lower willingness of EPs when the PTI was > 40 min.

The harmful effects of long TTI on neurological outcomes in OHCA cases were more pronounced in the short STI group than in the long STI group [38]. Moreover, in patients with refractory OHCA whose TTI was > 10 min, a longer STI was negatively associated with poor neurological outcomes [37]. STIs or TTIs are mutually interacting factors in the decision regarding the treatment of patients with refractory VF/VT. In our study, the difference in willingness according to the STIs was not significant regardless of the TTI. However, in intermediate TTI, the EPs showed varied willingness scores according to the STI. Additionally, the CPR quality during transportation may be less effective; therefore, the willingness to perform ECPR may be more strongly affected by TTI. When the STI was long, the willingness steeply decreased according to the TTI. In this case, a long STI could predict a poor prognosis, as shown in previous studies. Shortening of TTI may be based on the location of centers that provide advanced therapies for OHCA, for example, the regional designation of defined cardiac arrest centers. In an area without such systems, the decision to deploy advanced therapies may be variably applied, and it could be more dependent on individual doctors providing direct medical oversight.

The experimental vignette method can enable the estimation of unconfounded and context-dependent effects of explanatory vignette factors [17,18,21,39]. Furthermore, the experimental vignette method is used for the analysis of behaviors or attitudes to some context, and it can be utilized to train EPs to recognize cases wherein ECPR should be administered, if proper guidelines are available [40,41]. Therefore, the vignette method can be used to train doctors to reduce decision variability of direct medical oversight or maintain the regional OHCA care system.

The mean willingness scores of EPs for ECMO implementation were high (more than 75) across all vignettes. In total, 20 participants (37.0%), including six who responded with a 100% willingness score for all vignettes, showed willingness with an average score ≥ 90. Their responses did not show a significant difference for the time within the ischemic range of 45 minutes, i.e., the maximum prehospital time suggested in this study. Most EPs were confident that ECMO could be effective treatment for patient with refractory VF/VT. Further studies are needed to determine whether these responders show changes in willingness with longer ischemic time durations and to consider other factors, such as patient age, sex or comorbidities, that can affect their willingness.

This study had some limitations. We included only EPs and no other specialists who can deliver ECPR and post-resuscitation care. However, the vignette was based on an initial response in the emergency department; thus, EPs were the most appropriate investigation target. Second, only EPs were involved in the vignette development. Although ECPR requires multidisciplinary support and EPs do not often cannulate, they can recognize the patients who fit the inclusion criteria and initiate subsequent ECPR.

This study focused on the willingness to initiate ECPR. In the vignettes of this study, the prehospital time components, ATI and RTI, were not considered as factors. ATI and RTI factors correlate with outcomes in refractory VT/VF OHCA; however, these two components are strongly correlated with public attitudes toward OHCA rather than the EMS operation. ATI and RTI are related to both the probability of survival and EPs’ decisions regarding resuscitation. Therefore, to reduce the confounding effect, ATI and RTI were controlled in the vignettes and not considered in the factor analysis.

In conclusion, the willingness of EPs to implement ECPR remained high and appeared to be adversely affected by increasing STI, TTI, and total prehospital time.

## Supporting information

S1 File An example of the question to measure the EP’s willingness of ECPR application to the out-of-hospital refractory VT/VF patient. (PDF)

S2 File Dataset from the ECPR vignette survey. (XLS)
