# Supplementary material for: Analyzing willingness for extracorporeal cardiopulmonary resuscitation in refractory ventricular fibrillation
Source: PLoS One. 2023 Jan 26;18(1):e0281092. doi: 10.1371/journal.pone.0281092 (PMC9879451; doi:10.1371/journal.pone.0281092)

Supplementary material 1. An example of the question to measure the EP's willingness of ECPR application to the out-of-hospital refractory VT/VF patient

※ You should decide to apply ECPR to patients described in the following questions. Before answering the questions, please carefully review the following assumptions and answer each question.

[Assumptions]

1. The following assumes that the paramedic notifies the transfer of an out-of-hospital cardiac arrest patient while you are working in the emergency room.
2. It is assumed that the hospital where you work has a dedicated ECPR team and that ECPR is available 24/7.
3. The hospital where you work is the closest hospital to which paramedics can transfer, and the estimated transfer time in the questions is based on both distance and traffic conditions.

Case 1.

Paramedic: "Patient is a 55-year-old male. A co-worker witnessed him suddenly lose consciousness and collapse while eating lunch. A co-worker who witnessed this immediately called 911 and performed chest compressions according to the telephone instructions. About 6 minutes later, an ambulance arrived, and the AED recognized a shockable rhythm and performed defibrillation. We continued basic CPR and delivered two more defibrillations.
The total time spent on site was 11 minutes, and we just set off. VF is going on now. We will arrive at your hospital in about nine minutes."

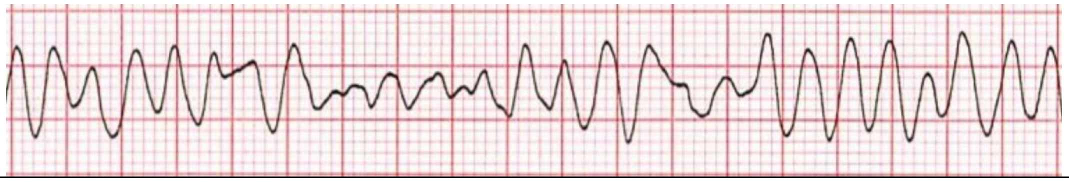

What is your willingness to activate the ECPR team for this patient?
Please move the indicator to a score between 0 and 100 points, with 0 points for 'no intention to activate the ECPR team' and 100 points for 'unconditionally activate the ECPR.'

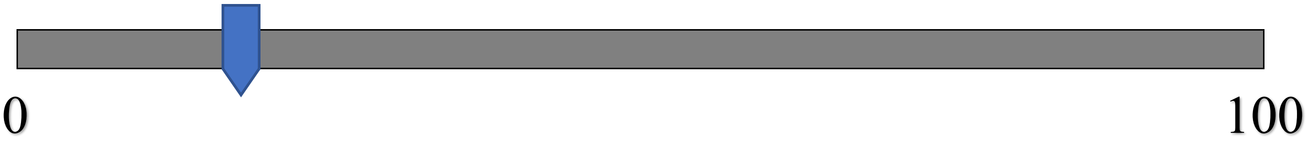

Supplement: S1 File — (PDF) [file pone.0281092.s001.pdf]
